# Metabolite–Flavor Profile, Phenolic Content, and Antioxidant Activity Changes in Sacha Inchi (*Plukenetia volubilis* L.) Seeds during Germination

**DOI:** 10.3390/foods10102476

**Published:** 2021-10-16

**Authors:** Kannika Keawkim, Yaowapa Lorjaroenphon, Kanithaporn Vangnai, Kriskamol Na Jom

**Affiliations:** 1 Department of Food Science and Technology, Faculty of Agro-Industry, Kasetsart University, Bangkok 10900, Thailand; k.kannika2252@gmail.com (K.K.); yaowapa.l@ku.th (Y.L.); kanithaporn.v@ku.th (K.V.); 2 Faculty of Science and Technology, Huachiew Chalermprakiet University, Bang Chalong 10540, Thailand

**Keywords:** antioxidant capacity, flavoromics, germination, metabolomics, *Plukenetia volubilis* L.

## Abstract

Sacha inchi seeds are abundant in nutrients such as linolenic acids and amino acids. Germination can further enhance their nutritional and medicinal value; however, germination time is positively correlated with off-flavor in germinated seeds. This study investigated the changes in the metabolite and flavor profiles and evaluated the nutritional quality of sacha inchi seeds 8 days after germination (DAG). We also determined their phenolic content and antioxidant activity. We used gas chromatography equipped with a flame ionization detector (GC-FID) and gas chromatography–mass spectrometry (GC-MS) and identified 63 metabolites, including 18 fatty acid methyl esters (FAMEs). FAMEs had the highest concentration in ungerminated seeds, especially palmitic, stearic, linoleic, linolenic, and oleic acids. Amino acids, total phenolic compounds (TPCs), and antioxidant activity associated with health benefits increased with germination time. At the final germination stage, oxidation products were observed, which are associated with green, beany, and grassy odors and rancid and off-flavors. Germination is a valuable processing step to enhance the nutritional quality of sacha inchi seeds. These 6DAG or 8DAG seeds may be an alternative source of high-value-added compounds used in plant-protein-based products and isolated protein.

## 1. Introduction

Sacha inchi (*Plukenetia volubilis* Linneo), from the Euphorbiaceae family, also known as inca peanut, wild peanut, or sacha peanut, is generally cultivated in many regions of the Peruvian Amazon [1]. Sacha inchi has significant potential economic value in cosmetic, pharmaceutical, and food industries, and has recently been introduced as an alternative crop in Thailand. In 2020, the global Sacha Inchi market volume was valued at 92 Million USD in 2020. However, it is expected to receive a higher market share with a CAGR of 4.7% to reach 125.8 million USD during 2021–2027 [2]. The increasing demand for vegan, organic products, protein-rich food products, and supplements, as well as its demand in the food and beverages industry and the cosmetics sector, is expected to push the sacha inchi industry growth [3]. At present, several commercial products have increased availability in particular oil, roasted seeds, and protein powder or flour.

Sacha inchi seeds are nutritionally superior as a good source of oil (~41%), protein (~27%), and bioactive compounds [4], such as polyphenolic compounds, phytosterols (stigmasterol and campesterol), and tocopherols. The oil is rich in unsaturated fatty acids (~93% of total lipids). Essential fatty acids in the seeds comprise 33% α-linoleic acid and ~51% α-linolenic acid [5]. The seeds contain high amounts of essential amino acids, such as isoleucine, valine, and tryptophan [6]. However, they also contain antinutritional components, such as saponins, tannins, and trypsin inhibitors [7,8], and are usually consumed roasted because of the astringency due to antinutritional components. The germination conditions of sacha inchi could reduce tannins and other antinutritional components and enhance the seeds’ nutritional quality [9].

Germination has been studied in many seed plants, such as soybean [10], barley [11], mung bean [12], sunflower [13], and mangosteen [14]. A germinating seed depends on reserve carbohydrates, proteins, and lipids synthesized and stored during seed growth. These compounds are synthesized during germination via glycolysis, the tricarboxylic acid (TCA) cycle, and amino acid metabolic pathways. Germination begins with water imbibition, food reserve mobilization, protein synthesis, and consequent radicle protrusion—the main food reserve for seedling development. The food reserves may be stored mainly in the endosperm (many monocotyledons, cereal grains, and castor) or cotyledons (many dicotyledons, such as peas and beans). The macronutrients (proteins, lipids, and carbohydrates) are enzymatically (amylases, proteases) hydrolyzed into simple units of food comprising sugars and amino acids. These dissolve in water and pass toward the growing epicotyl, hypocotyl, radicle, and plumule through the cotyledons [15]. Triacylglycerol (storage lipids) metabolism during germination has also been reported in sacha inchi. During germination, triacylglycerols are hydrolyzed into fatty acids by lipase via lipid metabolism. The released fatty acids undergo β-oxidation to generate adenosine triphosphate (ATP) as the major energy supply within the seeds [16].

However, germination time is positively correlated to off-flavor in germinated seeds. The activity of lipoxygenases (LOXs) on storage lipids in seeds can produce oxidation products, such as aldehydes and alcohols, leading to unpleasant beany, green, or rancid flavors [17]. The metabolites and flavor compounds that change during germination can be used as biomarkers according to the germination stages in sacha inchi.

Metabolomics is the scientific study of multiple small molecules in cells, tissues, plants, or foods. The metabolite profile contains valuable data for breeding-driven metabolic engineering of nutritionally important metabolites in plants. Metabolomics has been applied in plant and food science to study the effect of germination on mung bean [12] and barley [11] and the effect of processing and storage on Nam Dok Mai mango wine fermentation [18]. The metabolomics approach can be targeted or nontargeted. Nontargeted metabolomics can be used to simultaneously analyze hundreds or thousands of known and unknown metabolites. In addition, flavor metabolomics or flavoromics can be applied in various food products, focusing on the changes in the targeted and nontargeted flavor compounds. 

There is still a lack of research information on the germination of sacha inchi seed, as well as the metabolism of triacylglycerols during seed germination of sacha inchi [19]. Only some limited information of the changes of fatty acids during germination of sacha inchi was investigated without any report on other nutrients.

Considering the insufficient information about the changes of metabolites, flavor compounds, as well as total phenolic compounds and antioxidant activity during germination of sacha inchi, therefore, this study investigated the different metabolites and flavor components formed during germination in sacha inchi seeds using coupled metabolomics–flavoromics analysis. We also investigated the phenolic content and antioxidant activity at different germination times. In addition, we identified important biomarkers during germination in order to determine the best nutritional values and acceptability of foods and ingredients made from sachi inchi seeds in the future.

## 2. Materials and Methods

### 2.1. Chemicals

All chemical solvents and reagents for extraction and gas chromatography (GC) derivatization were high-performance liquid chromatography (HPLC) and analytical grade and were purchased from RCI Labscan Ltd. (Pathumwan, Bangkok, Thailand) and Sigma-Aldrich (St. Louis, MO, USA), respectively. All internal standards, including tetracosane (IS I), 5α-cholestane-3β-ol (IS II), phenyl-β-d-glucopyranoside (IS III), ρ-chloro-l-phenylalanine (IS IV), and dodecanoic acid ethyl ester, were standard grade and were purchased from Sigma-Aldrich. In addition, all reference standards for metabolomics and flavoromics, a C6–C30 n-alkane mixture used to measure linear retention indices, and 2,2-diphenyl-1-picrylhydrazyl (DPPH), 2,2-azobis(3-ethylbenzothialzoline-6-sulfonic acid) (ABTS), and Folin–Ciocalteu reagent were purchased from Sigma-Aldrich.

### 2.2. Seed Sample Preparation

Raw sacha inchi seeds were purchased from Kaeng Khro City, Chaiyaphum Province, Thailand in 2020. Healthy intact seeds were disinfected by soaking them in 1% (active ingredient) hypochlorite bleach solution for 15 min, followed by washing thrice with sterilized distilled water. Next, the seeds were soaked in ultrapure water at 30 °C for 12 h and then placed between wet sterilized tissue papers in a sterilized container and watered regularly every 12 h. The container was kept in an incubator at 30 °C and 80% humidity [19]. 

**Figure 1:**
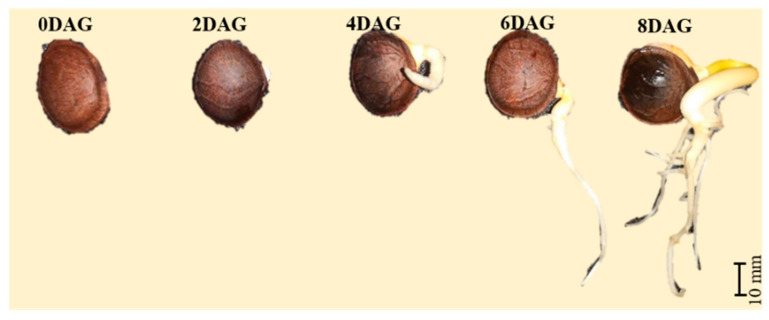
Germinated sacha inchi seeds at different germination days. DAG, days after germination.

Germination was defined by the appearance of an emerging radicle. The samples were kept for 2, 4, 6, and 8 days (Figure 1). The germinated samples were peeled and freeze-dried for 24 h using a Gamma 2-16 LSC freeze-drying machine (Martin Christ, Osterode am Harz, Germany), ground into a fine powder using a blender, and stored in an aluminum bag at −18 °C until analysis.

### 2.3. Nontargeted Metabolomics

Solid-phase extraction (SPE) and fractionation of samples were performed [12]. Briefly, lipids and polar compounds (sugars, sugar alcohols, acids, amino acids, organic acids, and amines) were extracted from the samples (100 mg) using SPE many times. Dichloromethane was added to elute the lipid fraction, and the polar fraction in the defatted sample was eluted using mixed solvents (80:20 *v*/*v* of methanol and deionized water [DI]). 

After adding 100 µL each of IS I and IS II solution to 500 µL of the lipid fraction, lipid transesterification was carried out in methanol, and consequently, lipids were separated into fractions using an SPE C18-LP cartridge (Vertical Chromatography Co., Ltd., Nonthaburi, Thailand). Fraction 1 contained fatty acid methyl esters (FAMEs) and hydrocarbons, and fraction 2 contained polar lipids (free fatty acids [FFAs], fatty alcohols, and sterols). All eluents were dried using a vacuum evaporator. The dried fraction 2 was silylated with n-methyl-n-(trimethylsilyl)trifluoroacetamide (MSTFA). 

Subsequently, 250 µL each of IS III and IS IV solution was added to the polar fraction to obtain fraction 3, 1 mL of which was dried and then silylated with n-trimethylsilylimidazole (TMSIM). To obtain fraction 4, 1 mL of the polar extract was evaporated to dryness and 100 µL of the silylating agent MSTFA was added to the oximated sample and silylation performed at 70 °C for 15 min. 

To obtain acids, 500 µL of hexane and 300 µL of DI water were added to the sample. After vortex mixing, the oximated sugar in the upper phase was removed and the lower phase (amino acids and free organic acids) was collected and evaporated until dryness and then silylated with MSTFA. 

Finally, 1 µL of all four fractions was separately analyzed by gas chromatography equipped with a flame ionization detector (GC-FID; Hewlett Packard, Palo Alto, CA, USA) to characterize them. A DB-1 capillary column (60 m × 0.32 mm × 0.25 μm film thickness) with a 100% dimethylpolysiloxane stationary phase (Agilent Technologies, Santa Clara, CA, USA) was used to separate the components in the sample. Helium was used as the carrier gas at a constant flow rate of 1.8 mL/min. Splitless injection was performed at 280 °C. The oven temperature program started at 100 °C, then ramped up to 320 °C at 4 °C/min, and was held at 320 °C for 25 min. The detector temperature was set to 320 °C. 

### 2.4. Nontargeted Flavoromics

Flavor compounds in sacha inchi seeds were investigated and measured using 6890N GC coupled with a time-of-flight mass spectrometer (Leco Corp., St. Joseph, MI, USA). Dodecanoic acid ethyl ester was used as an internal standard, and a group of n-alkanes (C6–C30) were used as standards to determine the retention index. Autosampler headspace solid-phase microextraction (HS-SPME) was performed to extract all volatile compounds from the sample [20]. 

To extract flavor compounds, ~3 g of the sample was put in a 20 mL headspace vial and 1 µL of dodecanoic acid ethyl ester (1 µg/mL in methanol) and 1 g of NaCl were added before sealing the vial cap. The sample was automatically equilibrated at 70 °C for 10 min. The volatile compounds were adsorbed on 50/30 µm divinylbenzene/carboxen/polydimethylsiloxane (DVB/CAR/PDMS) fiber at 70 °C for 30 min. Then, the fiber was desorbed into the GC injection port at 250 °C for 5 min in splitless mode.

GC 6890N coupled with a time-of-flight mass spectrometer (Leco Corp.) was used to measure the volatile compounds in the sample. A Stabilwax fused silica column (30 m × 0.25 mm × 0.25 μm film thickness) with a cross-bond polyethylene glycol stationary phase was used to separate volatile compounds. The temperature program was started at 40 °C for 5 min, ramped up to 225 °C at 4 °C/min, and then held for 15 min. For MS, the ion source temperature was set to 200°C and the transfer line temperature to 225 °C. Mass spectra were investigated at −70 eV in the m/z range of 35–500 atomic mass units (amu). Identification and relative concentration of flavor compounds were performed using the ChromaTOF-GC software v4.50.8.0. 

### 2.5. Determination of Total Phenolic Compounds

In the extraction method of Atala et al. (2009) with slight modifications [21], 2.5 g of the sample were extracted with 25 mL of 75:25 *v*/*v* acetone and DI water solution and shaken in a water bath at room temperature for 90 min. Then, the extract was centrifuged at 2000× *g* for 15 min, and the supernatant was collected and kept in a brown vial at −20 °C to evaluate the total phenolic compounds (TPCs) and antioxidant activity.

The TPCs were determined using the protocol of Chirinos et al. (2016) with slight modifications [22]. Briefly, 500 µL of the sample extract was mixed with 1250 µL of Na_2_CO_3_ (7.5% *w*/*v*) and 250 µL of Folin–Ciocalteu reagent and reacted for 30 min at room temperature. Next, absorbance was measured with a spectrophotometer at 755 nm wavelength against a blank in 500 µL of DI water, 1250 µL of Na_2_CO_3_, and 250 µL of Folin–Ciocalteu reagent. The results are expressed as gallic acid equivalents (mg GAE/g sample).

### 2.6. Determination of Antioxidant Activity 

DPPH and ABTS assays were performed to evaluate the antioxidant activity using a spectrophotometer. The 0.1 mg/mL (75:25 *v*/*v* acetone and DI water solution) of extracted solution was used to determine antioxidant activity. Ascorbic acid was used as positive control. The antioxidant activity was expressed in terms of radical scavenging activity by the following equation:

Radical scavenging activity (%) = [(A_0_ − A_s_)/A_s_] × 100

where, A_0_ is absorbance of blank, and A_s_ is absorbance of sample extract.

In DPPH assay, as described by Brand-Williams et al. (1995) [23] with modifications, 200 µL of the sample extract was reacted with 2.8 mL of DPPH (0.1 mM in methanol) solution. After incubation for 30 min in the dark, absorbance was measured at 517 nm wavelength against a blank (200 µL of methanol in 2.8 mL DPPH solution). 

In the ABTS assay, as described by Re et al. (1999) [24] with modifications, ABTS·+ solution was prepared by mixing ABTS solution (7 mmol/L) with K_2_S_2_O_8_ solution (2.45 mmol/L) in a 1:1 volume ratio and stored for 12–16 h in the dark. ABTS·+ solution was diluted with ethanol in a 1:50 volume ratio to an absorbance of ~0.7 ± 0.02 at 734 nm wavelength. Then, 1 mL of ABTS·+ solution was added to 10 µL of the sample extract and reacted for 15 min in the dark. Absorbance was measured at 734 nm wavelength against a blank (10 µL of DI water in 1 mL ABTS·+ solution). 

### 2.7. Statistical Analysis

The collected data were measured at least twice. The identical peak areas of metabolites were integrated using the HP ChemStation A.06.03 program (Hewlett Packard) and identified using the reference standards comparison method. The relative quantification was done by normalizing the corresponding peak areas to the peak area of the internal standard of each fraction and dividing the value by the weight of the extracted sample. The mass spectra of detected compounds were identified by matching mass spectra with NIST mass spectral database version 2.0 (National Institute of Standards and Technology, Gaithersburg, MD, USA). The retention time index (RI) of each volatile compound was calculated using the retention time of the n-alkanes series (C6–C30) as a reference. The relative concentration of identified compounds was quantified using the internal standard method. Dynamic changes in all metabolites and flavor compounds during germination were explained by principal component analysis (PCA), heat plots, and Spearman’s rank correlation. PCA was performed using XLSTAT base software. Correlation network analysis using nonparametric Spearman’s rank correlation of all metabolites and flavor compounds was performed at a significance level of *p* ≤ 0.05 in the correlation/association test mode in XLSTAT.

## 3. Results and Discussion

### 3.1. Integrated Metabolomics and Flavoromics of Germinated Sacha Inchi Seeds

The germination percentage of sacha inchi seeds was ≥80%. The seeds began germinating after 2 days and reached the maximum germination percentage at 8 days. Metabolomics identified 63 (~65%) of a total of 95 metabolites at 0DAG–8DAG, similar to comparable metabolite profiling studies on mung bean [12] and soybean [25]. The 63 metabolites were categorized into 4 groups: 18 FAMEs (16 FAMEs and 2 hydrocarbons), 17 polar lipids (10 FFAs, 2 fatty alcohols, and 5 phytosterols), 6 sugars (3 organic sugars and 3 sugar alcohols), and 22 acids (17 amino and 5 organic acids). Flavoromics identified 35 (23%) of a total of 150 flavor compounds, similar to germinated brown rice (41 volatile compounds identified at 0DAG–5DAG [17]). The 35 flavor compounds were categorized into 7 groups: 9 aldehydes, 12 alcohols, 2 ketones, 8 volatile acids, 2 lactones, pyridine, pyrazine, and 2-methoxy-3-(1-methylethyl). 

**Figure 2:**
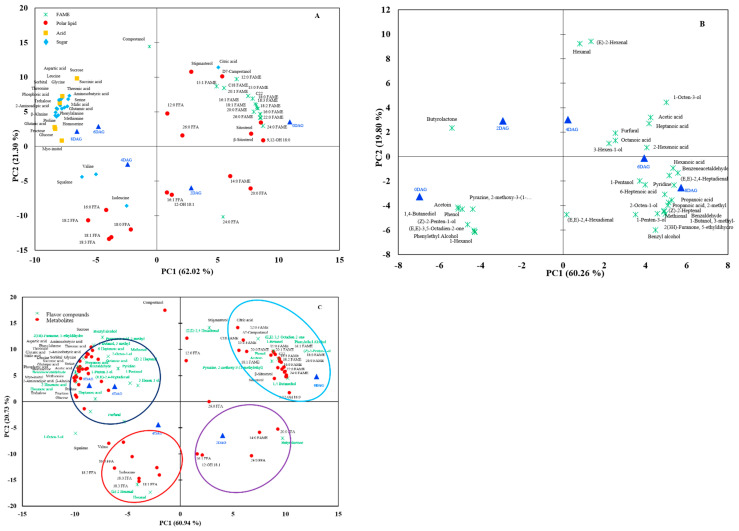
Biplot of principal component analysis from metabolites (**A**), flavor compounds (**B**), and metabolites and flavor compounds (**C**) in sacha inchi seeds at different germination times (*p* ≤ 0.05). FAME, fatty acid methyl ester; FFA, free fatty acid; DAG, days after germination.

Figure 2 shows the PCA biplot and loading of all identified metabolites and flavor compounds at different germination times. Polar and nonpolar metabolites were major contributors to the separation along principal component (PC) 1 (Figure 2A). The flavor compounds were prominently arranged at 4DAG, 6DAG, and 8DAG, explaining 75% of the variation (Figure 2B). The PCA biplots of metabolites and flavor compounds displayed four distinct clusters along with the PC (Figure 2C). The first two PCs explained 81.67% of the total variation at 0DAG–8DAG, PC1 and PC2 demonstrated eigenvalues of 60.330 and 20.518, respectively, and explained 60.94% and 20.73% of the total variation, respectively. Metabolites and flavor compounds were categorized based on the difference in the germination times into four clusters. The differences between ungerminated seeds (0DAG) and germinated seeds could be generally described on PC1. The 0DAG seeds relied on cluster 1 (17 FAMEs, 5 phytosterols, and 8 flavor compounds), 2DAG on cluster 2 (1 FAME, 5 FFAs, and 1 flavor compound), 4DAG on cluster 3 (5 FFAs, 3 amino acids, and 4 flavor compounds), and 6DAG and 8DAG on cluster 4 (all sugars, most of the acids, and 20 flavor compounds), indicating that most of the FAMEs and FFAs decrease, while most of the sugars, amino acids, aldehydes, alchohols, and volatile acids increase throughout germination in sacha inchi.

**Figure 3:**
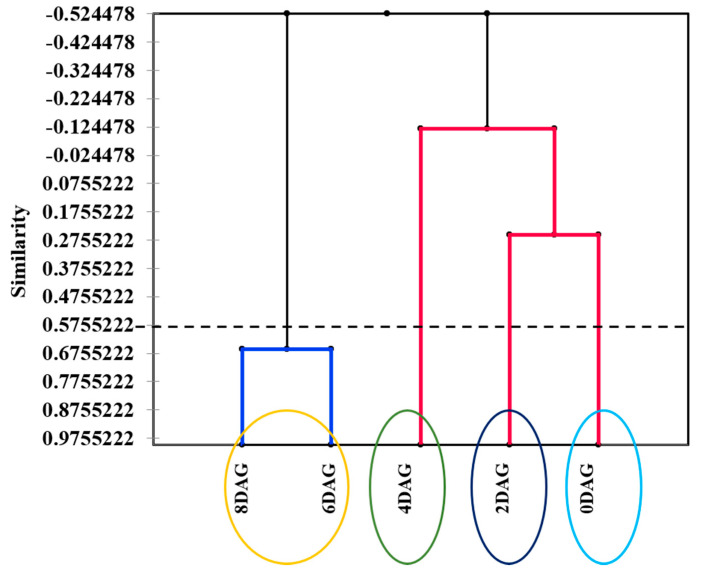
Dendrogram (similarity mode of agglomerative hierarchical clustering) of germinated sacha inchi seeds at different germination times (*p* ≤ 0.05). DAG, days after germination.

To confirm PCA grouping, agglomerative hierarchical clustering analysis in similarity mode was performed based on 0DAG–8DAG (Figure 3). The dendrogram confirmed the similarity among the germination stages, differentiated into four groups: group 1 included ungerminated seeds (0DAG); group 2, 2DAG seeds; group 3, 4DAG seeds; and group 4, 6DAG and 8DAG seeds. The coupled metabolomics–flavoromics of 0DAG was closer to that of 2DAG, whereas that of 4DAG was closer to that of 6DAG and 8DAG, indicating that metabolic and flavor components change more rapidly at the final compared to the early stage of germination.

**Figure 4:**
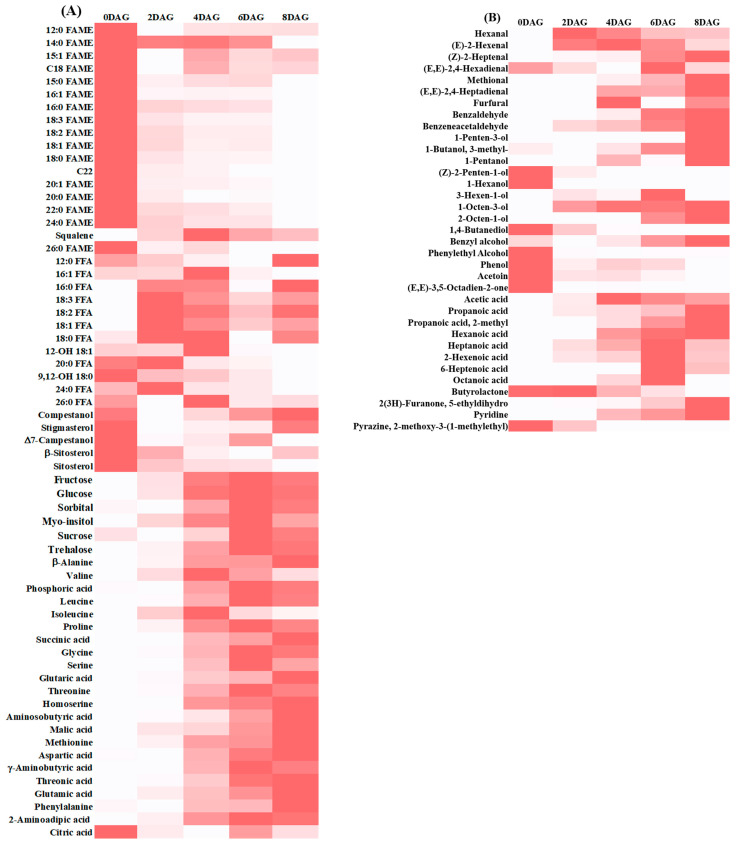
Heat plots of metabolites (**A**) and flavor compounds (**B**) in sacha inchi seeds at different germination times. FAME, fatty acid methyl ester; FFA, free fatty acid; DAG, days after germination.

Heat map analysis was used to represent dynamic changes in specific compounds by relative concentrations at each germination time (Figure 4). Figure 4A shows a heat map of all metabolites. The FAME contents were higher on day 0 and significantly decreased during germination. Linolenic (50%), linoleic (36%), and oleic (11%) acids were most abundant in the lipid fraction, followed by stearic (4%) and palmitic (0.25%) acids at 0DAG–8DAG. Compared to ungerminated sacha inchi seeds, the palmitic acid content decreased by ~65.6-fold, followed by stearic (13.6-fold), linoleic (12.3-fold), linolenic (11.7-fold), and oleic (4.9-fold) acids. A decrease in FAMEs has also been reported in germinated rice [26], barley [11] and mung bean [12]. During germination, triacylglycerols are hydrolyzed by lipase into FFAs and glycerol, and then hydrolyzed fatty acids are degraded into glyoxysomes by β-oxidation. Fatty acids are oxidized to carbon dioxide and water, producing energy for the germination and biosynthesis of new compounds. Therefore, palmitic, stearic, linoleic, linolenic, and oleic acids can be used as energy sources during embryo development in germinating sacha inchi. In addition, LOX is the main pathway to mobilize lipids during germination [27].

We found 10 FFAs, 2 fatty alcohols, and 5 sterols during germination. The FFA content was the highest at 2DAG, and then significantly dropped followed by a slight increase. Linolenic acid had the highest concentration at 2DAG (~27-fold), followed by oleic (13-fold), and linoleic (9-fold) acids, compared to ungerminated sacha inchi seeds. The increase in major FFAs at the beginning stage in the present study was similar to related research. Chandrasekaran et al. (2015) [19] reported that the major unsaturated fatty acids content increased at the beginning stage (3–10 days after germination), followed by a slight decrease during the germination course of sacha inchi. Under the different conditions on germination such as temperature, moisture, and germination time may influence lipid degradation rates [11] in germinated sacha inchi. At the beginning of germination, triacylglycerols are hydrolyzed by lipases into FFAs. Then, the FFAs are distributed into the mitochondrial matrix and degraded to acetyl-CoA by oxidation, which is then processed in the Krebs and glyoxylate cycles [28]. The fatty alcohol content in germinated sacha inchi seeds showed a relatively small change compared to ungerminated seeds: 9,12-OH 18:0 showed the highest concentration at 0DAG and 12-OH 18:1 at 4DAG. The changes in sterol levels during germination were relatively small, with a higher concentration at 0DAG than at other days. The ∆7-campestanol and sitosterol levels significantly changed during germination, similar to germination in wheat [29].

Six sugars (fructose, glucose, sorbitol, myo-inositol, sucrose, and trehalose) were found. Between 0DAG and 2DAG, the disaccharide sucrose level rapidly dropped, but drastically increased later. Similar results were seen in germinating mangosteen [14]. Fructose, glucose, sorbitol, myo-inositol, and trehalose increased up to 6DAG and gradually declined later. The fructose content changed from 0DAG to 8DAG. The increase in mono- and disaccharides during germination might suggest that carbohydrates and sugars are hydrolyzed to simple molecules at the beginning of germination to provide germinating energy for sacha inchi [30]. During seed growth, sacha inchi could continue to collect soluble sugars until late maturation. The consumption of soluble sugars during initial germination might have been affected by glycolysis and the TCA cycle. In organic mung bean, sugar accumulation during germination might be due to the degradation of starch in the endosperm via glycolysis, which provides the main energy source for germination [31]. 

During germination, enzymes (proteinases, oligopeptidase, peptidase, and aminopeptidases) hydrolyze storage proteins in the endosperm into amino acids [15]. In germinating rice, the protease pathway is the main pathway for degradation of storage proteins into free amino acids [32]. We identified 22 metabolic acids in germinating sacha inchi seeds, including 17 amino and 5 organic acids. Amino acids increased up to 6DAG, except for valine and isoleucine, which reduced at 4DAG. Glycine, isoleucine, proline, and leucine levels changed from 0DAG to 8DAG. Some organic acids (phosphoric, succinic, malic, and 2-aminoadipic acids) significantly increased during germination, while citric acid decreased. Citric and malic acids are major organic acids with high abundance in the TCA cycle. A similar trend of citric acid decrease and malic acid increase is seen in barley [33]. The increase in succinic acid during germination, an intermediate product of the TCA cycle, can improve the TCA cycle rate [31]. 

Figure 4B presents changes in volatile compounds in sacha inchi seeds at different germination times. A number of volatile compounds developed during germination, which may be related to the macronutrient composition of the seeds. Macronutrients such as starch and protein have a strong affinity to flavor compounds and can trap them through reversible hydrogen bonding and hydrophobic interactions, holding a higher polarity aroma than inclusion complexes. During germination, starch and protein are hydrolyzed into small molecules by α-amylase and protease, respectively. Trapped flavor compounds can be released throughout germination [34,35,36]. We identified 35 volatile compounds, ~25.7% at 0DAG and ~60% at 6DAG and 8DAG. The largest groups were alcohols (34%), aldehydes (25.7%), and volatile acids (22.8%). At 0DAG, acetoin and (E,E)-3,5-octadien-2-one displayed the highest concentration, which contributed woody/creamy and earthy/green pepper odors. The highest levels of hexanal, (E)-2-hexenal, and 1-octen-3-ol developed at 4DAG, associated with green pea, grass, and mushroom odors. At 6DAG and 8DAG, 6 alcohols, 5 aldehydes, and 8 volatile acids were detected: 1-penten-3-ol, 1-pentanol, 3-hexen-1-ol, (Z)-2-heptenal, and 2-octen-1-ol are associated with green, beany, and grassy odors, providing rancid and off-flavors. Aldehydes and alcohols are derived from LOX activity and oxidation of fatty acids and generally cause rancidity [37]. These compounds are formed during LOX-mediated degradation of polyunsaturated fatty acids [38,39] and are expected to directly correlate with the quality attributes of final germinated seeds. The observed acidity of germinated sacha inchi seeds could be a significant technical difficulty in producing germinated products, mainly due to the increase in volatile acids at the final stages of germination. Germinated brown rice [17] and germinated chickpea, lentil, and yellow pea [9] also show increased hexanoic, heptanoic, octanoic, and nonanoic acids at the final stages of germination; however, these have a low odor threshold (<1). The changes influence this phenomenon in lipid degradation in sacha inchi seeds during germination [40]. Upon germination, the concentration of certain pleasant flavors in sacha inchi seeds increased, such as a soy-sauce-like odor and a peach flavor, which indicated furfural and 2(3H)-furanone,5-ethyldihydro, respectively. 

**Figure 5:**
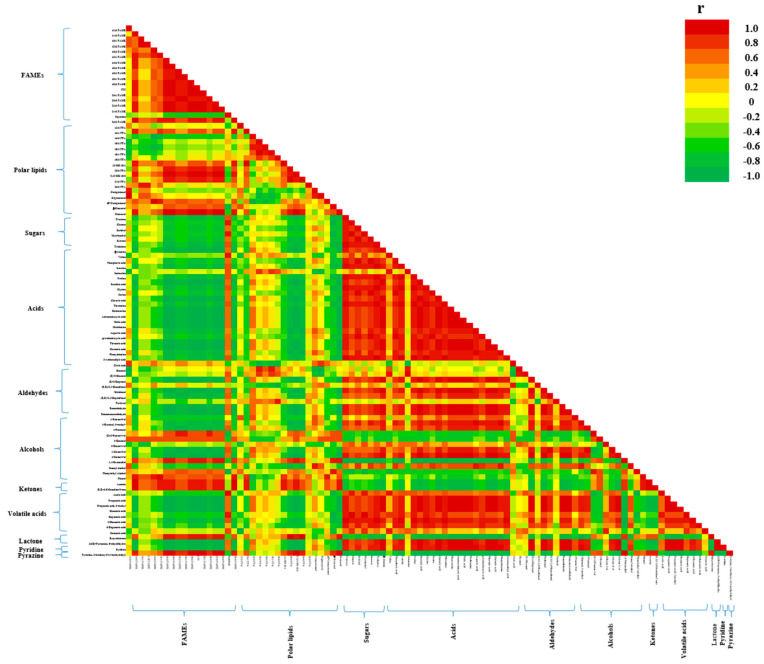
Lower triangular heat map represents pairwise correlation analysis between metabolites and flavor compounds during germination of sacha inchi seeds. Each square represents the Spearman’s rank correlation coefficient at a significance level of *p* ≤ 0.05. Orange-red, strong positive correlation (r > 0.7); green, strong negative correlation (r < −0.7). FAME, fatty acid methyl ester; FFA, free fatty acid.

Figure 5 shows the relation of all metabolites and flavor compounds during germination of sacha inchi seeds. Flavor compounds were mainly related to the lipid composition. FAMEs had a strong positive correlation (r > 0.7) with alcohols, ketones, butyrolactone, and pyrazine-2-methoxy-3-(1-methylethyl). However, FAMEs had a strong negative correlation (r < −0.7) with aldehydes, volatile acids, 2(3H)-furanone,5-ethyldihydro, and pyridine. In general, lipolysis and lipid oxidation produce rancidity and sourness attributed to aldehydes, alcohols, and volatile acids during germination [41]. During germination, volatile acids are formed via the lipolysis pathway. Aldehyde and alcohol formation during germination is correlated with the oxidation of oleic, linoleic, and linolenic acids as the main substrates through LOXs. These aldehydes and alcohols provide rancid and off-flavors to germinated sacha inchi seeds [42]. 

Coupled metabolomics–flavoromics analysis identified 10 metabolites (palmitic acid, stearic acid, oleic acid, linoleic acid, linolenic acid, fructose, glycine, isoleucine, proline, and leucine) and 7 flavor compounds (acetoin, (E,E)-3,5-octadien-2-one, hexanal, (E)-2-hexenal,1-octen-3-ol,2-octen-1-ol, and hexanoic acid) as biomarkers of the nutritional quality of ungerminated and germinated sacha inchi seeds. Palmitic, stearic, linoleic, linolenic, and oleic acids were the most abundant fatty acids, and the highest concentration of acetoin and (E,E)-3,5-octadien-2-one was found at 0DAG. At 4DAG, excess aldehydes and alcohols (hexanal, (E)-2-hexenal, and 1-octen-3-ol) were produced, which might be considered undesirable. At 6DAG and 8DAG, the highest level of sugars and amino acids was found, which could be applied as food ingredients or plant-based protein. The highest concentration of fructose, glycine, isoleucine, proline, and leucine can be used as a biomarker to identify the germinated seed quality. In addition, the changes in metabolites and flavor compounds during germination are also significantly correlated with biological activities [43,44].

### 3.2. Changes in TPCs and Antioxidant Activity during Germination

**Figure 6:**
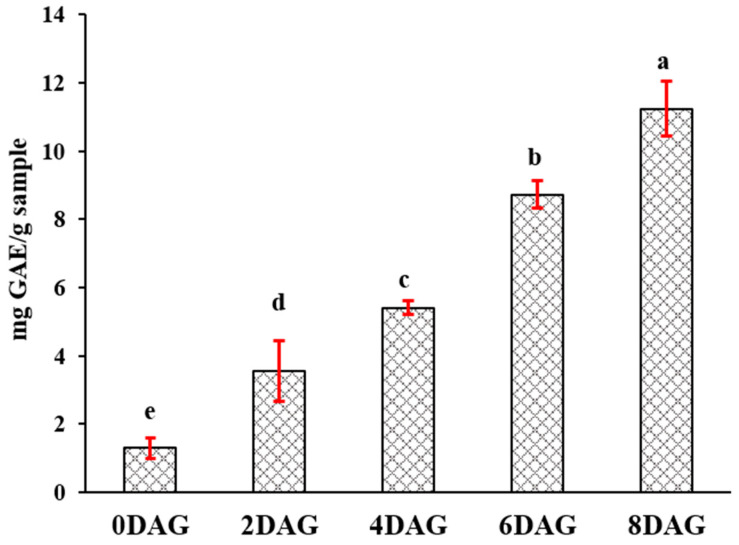
Total phenolic compounds (TPCs) of sacha inchi seeds at different germination times (*p* ≤ 0.05). DAG, days after germination. Means followed by different letters are significantly different (*p* ≤ 0.05) according to the Duncan’s new Multiple Range Test.

Germination relies on an effective method of achieving high concentrations of bioactive compounds. Phenolic contents also increase, apart from nutrition level changes. The dynamic changes in TPCs in germinated sacha inchi seeds are shown in Figure 6. TPCs gradually increased from 0DAG to 8DAG. The impact of germination on TPCs has been studied in edible seeds, such as beans and cereal grains. Germination can continuously collect soluble phenolics in germinated edible seeds compared with ungerminated seeds [45,46,47]. This collection could be associated with the shikimate pathway. Phenylalanine, as an intermediate metabolite in polyphenol metabolism, increased during germination. After the seeds absorb water, hydrolytic enzymes are activated and macromolecules (biopolymers, carbohydrates, and proteins) break down into small molecules in the endosperm, followed by an increase in simple sugars and free amino acids. In addition, bound phenolic compounds conjugated with the cell wall are also released [48]. Germination can also induce oxidative tension in a plant, producing more enhanced phenolics during germination for plant antioxidant protection [49]. In sacha inchi seed germination, TPCs were higher at 8DAG than at 0DAG. In addition, the polyphenol enrichment at 8DAG may be related to the formation of off-flavor compounds in sachi inchi seeds [44].

**Figure 7:**
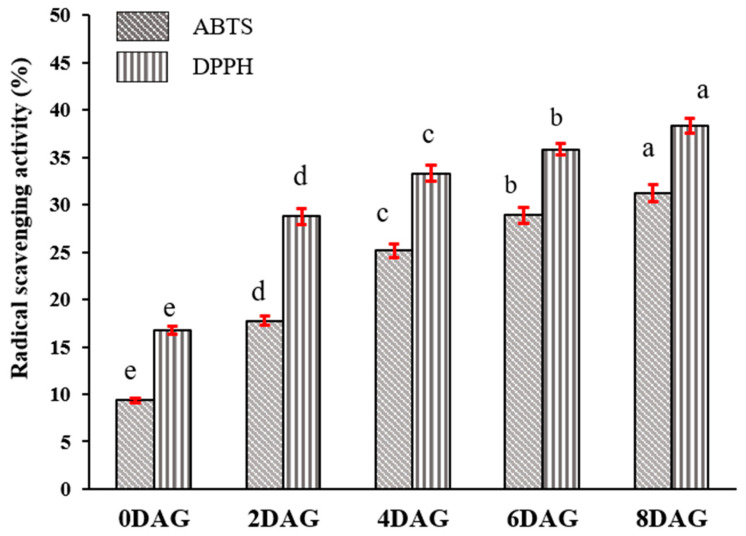
Antioxidant activity (ABTS and DPPH assays) of sacha inchi seeds at different germination times (*p* ≤ 0.05). ABTS, 2,2-azobis(3-ethylbenzothialzoline-6-sulfonic acid; DPPH, 2,2-diphenyl-1-picrylhydrazyl; DAG, days after germination. Means followed by different letters are significantly different *p* ≤ 0.05) according to the Duncan’s new Multiple Range Test.

In contrast, changes in the phenolic content were the main contributor to the antioxidant activity variation during germination [50]. The antioxidant activity of sacha inchi seeds at different germination times was evaluated by DPPH and ABTS assays (Figure 7). Germination significantly enhanced the antioxidant activity compared with nongerminated seeds. The antioxidant activity gradually increased from 0DAG to 8DAG up to 3.6- and 3-fold by DPPH and ABTS assays, respectively, and was parallel to the phenolic content, which, exhibiting a significant antioxidant potential, could be improved during germination. In addition, results showed that the highest value of the antioxidant activity on the germinated sacha inchi was stronger than the positive control of ascorbic acid at dosage of more than 10 and 20 µg/mL for DPPH and ABTS assays, respectively. Phenolics can be freed during germination, and some metabolic activities might improve due to the oxidative signaling in germinated seeds and generate energy for the developing plant. The increase in antioxidant activity might be associated with de novo synthesis and dynamic changes in phenolic compounds via phenylpropanoid biosynthesis. Germination can increase the antioxidant activity in edible seeds, such as jack bean, chick pea, soybean, kidney bean, and mung bean [48], attributed to the increase in some antioxidant components during germination, such as phenolic compounds, phytosterols, and vitamins. 

## 4. Conclusions

Coupled metabolomics and flavoromics can be used to identify the main metabolites and flavor compounds that change in sacha inchi seeds during germination in order to determine the best nutritional values and guide the improvement of pharmacological products or functional foods made from sacha inchi seeds. In particular, the later stage (6–8 DAG) should be applied as novel alternative plant-based protein, functional foods, and dietary supplements, because they contain significant amounts of amino acids and higher antioxidant activity. FAMEs (palmitic, stearic, linoleic, linolenic, and oleic acids) correlate with ungerminated seeds. Sugars and amino acids (fructose, glycine, isoleucine, proline, and leucine) correlate with 8DAG seeds. Flavor compounds acetoin and (E,E)-3,5-octadien-2-one have the highest concentration in ungerminated seeds. Hexanal, (E)-2-hexenal, and 1-octen-3-ol have the highest concentration at 4DAG and 2-octen-1-ol and hexanoic acid at 8DAG. The changes in these flavor compounds are biomarkers of flavor indicators in sacha inchi seeds. Amino acids, organic acids, TPCs, and antioxidant activity are higher in germinated sacha inchi seeds than in ungerminated seeds. These findings could help in understanding nutritional changes during germination. 

## Data Availability

Not applicable.
